# News and Notes

**Published:** 1907-01

**Authors:** 


### NEWS AND NOTES.

Flat-foot is another cause of pains in the leg or thigh.—*American Journal of Surgery.*

Long pauses between attacks of gastric or abdominal pain speak in favor of cholelithiasis.

Bronchietasis is not seldom complicated by brain abscess.—*American Journal of Surgery.*

Influenza is very prevalent in London, particularly in the fashionable quarters of the city.

Tuberculosis and cholelithiasis are only very rarely associated.—*American Journal of Surgery.*

The oldest existing European university is that of Bologna, which in 1888 celebrated its 800th anniversary.

A Cancer Research Fund has recently been established in Paris by an endowment of $20,000 from Baron Rothschild.

The silver-fork deformity is by no means necessary to the diagnosis of Colles' fracture.—*American Journal of Surgery.*

When palpating the common bile duct for stone ,make sure that a suspected calculus is not a gland.—*American Journal of Surgery.*

It is a peculiar fact that many of the cases of tumor of the bladder occur among workers in anilin dyes.—*American Journal of Surgery.*

If an incised wound in the soft parts does not heal as readily as it should, examine the urine for sugar.—*American Journal of Surgery.*

In cases of pain in the hip of doubtful origin, examination of the kidney regions may discover the cause.—*American Journal of Surgery.*

---

In catarrhal icterus the pulse is usually slow; in jaundice from cholelithiasis this is usually not the case.—*American Journal of Surgery.*

---

A mass protruding from the rectum of an infant or child may be an intussusception and not a mere prolapse.—*American Journal of Surgery.*

---

A pulsating tumor of the osilium (endothelioma, sarcoma) may easily be mistaken for a gluteal aneurism.—*American Journal of Surgery.*

---

No operation for sterility in the female should be performed without first excluding sterility on the husband's part.—*American Journal of Surgery*

---

The perinephric space is a frequent site of mentastatic inflammation after furunculosis or other septic infection.—*American Journal of Surgery*

---

If a patient vomits coffee-ground material in which no lactic acid is present, one can almost always exclude carcinoma.—*American Journal of Surgery.*

---

In differentiating between gastric ulcer and gall-stone pains, the association of a chill usually points to cholelithiasis.—*American Journal of Surgery.*

---

In a tuberculous patient with supposed chronic appendicitis it is well to suspect tuberculous disease of the ileo-cecal valve—*American Journal of Surgery.*

---

In the performance of the radical operation for breast carcinoma it is important to avoid ijury to the periosteum of the ribs.—*American Journal of Surgery.*

Prof. D. M. R. Culbreth has resigned the chair of materia medica in the University of Maryland. The lectures are now given by Prof. Joseph E. Gichner.

---

Hemorrhage from the bladder may yield to irrigations with ice-cold water and with 1-10,000 adrenalin solution, successfully—*American Journal of Surgery.*

---

In the presence of large masses of glands in the epigastrium, especially on the right side, examine the testicles for new growth.—*American Journal of Surgery.*

---

Lumbar puncture must not be performed in cases of tumor of the brain. Sudden death has frequently happened in such cases.—*American Journal of Surgery.*

---

In an attack of cholelithiasis the vomiting as a rule is not attended by relief of pain; the contrary is true in ulcer of the stomach.—*American Journal of Surgery.*

---

Bandage knives cut best when they have a "saw edge," which is easily secured by sharpening them on a window sill or other rough stone.—*American Journal of Surgery.*

---

Sixty cases of leprosy were reported at the Congress on Leprosy held recently at Buenos Ayres, and it was stated that a mosquito was the transmitting agent of the disease.

---

After tracheotomy the air of the patient's room should be kept reasonably warm and moist. Draughts of cold air provoke much irritation.—*American Journa lof Surgery.*

---

If a thrombosing pile is opened before the clotting is complete, it is very apt to fill up again and may even become edematous and inflamed.—*American Journal of Surgery.*

---

Before putting an unconscious person to bed, the hot water bags should be removed or sufficiently covered to prevent the occurrence of a burn.—*American Journal of Surgerqy.*

In explornig for tumors of the brain, the best guide for determining an isolated hardness is the finger; the use of a needle is very deceptive.—*American Journal of Surgery.*

---

Americans are taking the lead in organizing an international association of medical museums to catalog specimens and enable duplicates to be exchanged or specimens loaned.

---

The board of trustees of the Carnegie Institution at a recent meeting in Washington appropriated $661,300 to aid in scientific research of various kinds during the year 1907.

---

If an undoubted case of ulcer of the stomach is associated with chills, in most cases it means that the ulcer is adherent to the spleen. —*American Journal of Surgery.*

---

If pressure in the right hypogastrium gives rise to a referred pain in the shoulder region, the offending area is probably the gall-bladder and not the pylorus.—*American Journal of Surgery.*

---

All cases of hernia in which there is a history of frequent urination should lead one to the suspicion that the hernial sac contains part of the bladder.—*American Journal of Surgery.*

---

In operations for suture of a fractured patella it is very important to sew the torn lateral ligaments of the joint. These aid largely in the support of the joint.—*American Journal of Surgery.*

---

A sudden desire for sharp, sour and spicy articles of food in a middle-aged or elderly person is often the first symptom of a beginning gastric carcinoma.—*American Journal of Surgery.*

---

The appearance of pus in the breast of a woman who is not, or has not recently been nursing, is suspicious of some unusual form of infection, e. g., tuberculosis.—*American Journal of Surgery.*

---

Repeated attacks of coughing after tracheotomy may mean irritation of the posterior wall of the trachea by the tube; change the length or shape of the canula.—*American Journal of Surgery.*

A hematoma may be produced in the calf muscles by direct or indirect violence that the patient may pay little attention to at the time or even fail to recall.—*American Journal of Surgery.*

---

In the presence of a breast infection that fails to heal within a reasonable time after appropriate incision and dressings, it is well to think of local tuberculosis.—*American Journal of Surgery.*

---

In the presence of a breast infection that fails to heal within a reasonable time after appropriate nicisions and dressings, it is well to thin kof local tuberculosis.—*American Journal of Surgery.* . .

---

In the aged pain and disability in the arm after traumatism demand especial care in examination of the shoulder. Fracture of the head of the humerous is often overloooked.—*American Journal of Surgery*

---

Do not be too hasty in resecting a strangulated loop of intestine. It is remarkable how frequently such loops become viable after long continued applications of hot saline solution.—*American Journal of Surgery.*

---

Fractures of the head of the radius are probably more common than generally supposed, being overlooked frequently because of the absence of the ordinary signs of fracture.—*American Journal of Surgery.*

---

It is wrong to perform any radical operation for an ulcer of the tongue without preliminary microscopical examination. Clinical symptoms, no matter how typical, are often misleading.—*American Journal of Surgery.*

---

Aluminum instruments should not be boiled in soda solution, like other instruments. They are to be sterilized by boiling in plain water or by passing them through an alcohol or Bunsen flame.—*American Journal of Surgery.*

---

During the performance of a hernia operation it is often helpful for the anesthetist to allow the patient to react sufficiently to strain into view a sac that has slipped back into the abdomen.—*American Journal of Surgery.*

If a peculiar looking mass is found at the inner side of the ring in the course of an operation for inguinal hernia, do not incise or dissect it before convincing yourself that it is not the bladder.—*American Journal of Surgery.*

---

A bichloride of mecrury dressing should never be applied on an area of skin on which tincture of iodin has been recently painted. An idodid of mercury is formed, which is highly irritating.—*American Journal of Surgery.*

---

As a final cleansing step after curettage of the uterus it is well to introduce, and at once withdraw, a packing of gauze. This brings out with it fragments of tissue not washed out by the irrigation.—*American Journal of Surgery.*

---

When the openings of the Bartholinian glands appear as two sharply defined red spots, an antedating inflammation may be diagnosed with certainty, and in a great majority of instances a latent gonorrhea is present.—*American Journal of Surgery.*

---

In persons of middle age presenting gastric symptoms, the diagnosis of cancer should not be excluded because the symptoms have had a sudden onset. Such an onset occurs in a fair proportion of cases.—*American Journal of Surgery.*

---

In light narcosis the pupils may dilate reflexly from operative manipulations. This, of course, is not to be confused with the sudden extreme dilation that occurs when the narcosis has been carried too far.—*American Journal of Surgery.*

---

In France there were eleven hundred less medical students in 1906 than there were in 1895, not counting foreign students whose numbers had diminished 46.8 per cent. In the United States there was a decrease in 1906 of about 25 per cent.

---

The employment of adrenalin as an application with cocain to the mucous membrane of the cheek, *e. g.*, for the excision of a leukoplakic ulcer, is not to be advised. There may be severe secondary hemorrhage.—*American Journal of Surgery*

Bleeding from the capillary hemorrhoids high in the rectum usually yields to injections of cold water, or a cold solution of tannic acid. In these cases, however, it is important to exclude the presence of an ulcer further up.—*American Journal of Surgery.*

---

If there is repeated vomiting and the pateint shows some evidences of collapse, after a laparotomy, especially after operations in the gastric region, examine for separation of the wound and prolapse of the abdominal contents.—*American Journal of Surgery.*

---

The early reappearance of fluid after tapping a hydrocele does not necessarily mean that the operation has been a failure. It may be but an inflammatory reaction, subsiding spontaneously or under the application of unguentum iodi.—*American Journal of Surgery.*

---

A light-bearing cystoscope is a handy instrument for the non-specialist to use for transillumination of the accessory sinuses of the nose. Place the tip of the instrument in the patient's mouth and let him close his lips firmly.—*American Journal of Surgery*

---

The National Sanitarium Association of Canada has established a new magazine, known as *Canadian Outdoor Life.* Its purpose will be to advocate the outdoor treatment of tuberculosis, and it will also have useful educational matter on the hygiene of living.

---

Persistent pains in the leg may be due to obliterating endarteritis. This occurrs occasionally in young men and often goes on to the production of gangrene. Both syphilis and excessive smoking are suspected as etiological factors.—*American Journal of Surgery*

---

Professor Neisser (Breslau) has been commissioned by the German imberial authorities to pursue his researches on syphilis, and will accordingly tart on a second expedition to Batavia. He will be accompanied by Drs. Halberstadter von Prowazek, Bruck, and Siebert.

---

Dr. John A. Ouchterlony, of Louisville, who died recently, bequeathed $100,000 for the foundation of a tuberculosis hospital in Louisville. The will was contested, but a compromise was reached, and the bequest is available for the purpose mentioned.

In the progress of a cholecystectomy, if a stone slips away after cutting through the cystic duct and cannot be found, no great anxiety need be felt, for the stone usually comes away spontaneously in the subsequent discharge.—*American Journal of Surgery.*

---

The Kentucky State Board of Health has prosecuted and is reported have obtained a verdict with a fine of $50 against John A. Taff, proprietor of the Louisville Sanitarium, an institution operated for the "cure of locomotor ataxia, paralysis and kindred diseases."

---

The Boston & Maine Railroad Company has put in commission, with headquarters at Boston, a new hospital car. Among the special features are easy springs, spacious bed, nurses' quarters and cabinet, kitchen, independent heating and lighting and wide folding side doors.

---

In acute (septic) osteomyelitis immediate operation is not too radical; in chronic osteomyelitis patient waiting is often not too conservative—the final expulsion of a sequestrum may be all that is necessary to effect spontaneous cure.—*American Journal of Surgery. ery.*

---

The question of raising the standard of entrance exminations of the medical colleges of North Carolina will be brought out at the forthcoming session of the State legislature. The faculties of the medical schools are reported to be in favor of making this much needed reform.

---

If a small child has been pulled by the arm and thereafter has disability in that member, attention should first be directed to the upper end of the radius. Here is one apt to find a subluxation of the head of the bone ("pulled arm") or an epiphysial separation.—*American Journal of Surgery.*

---

Nitrate of silver may be attached in full strength to the end of a probe, as for application in the middle ear, by heating the tip of the instrument and pressing it into the stick of caustic; a little of the latter will melt and form a bead on the probe when it cools.—*American Journal of Surgery.*

An ointmhent of beta-naphthol, 10; sulphur, 45; lard, 24; and green soap, enough to make 100 parts, is useful in removing gun-powder not too deeply situated in the skin. It must be employed cautiously, however, to avoid a destructive dermatitis.—*American Journal of Surgery.*

---

Dr. J. E. Atkinson, one of Baltimore's most prominent physicians, died November 24th, after a few days' illness with pneumonia. Aged 60 years. Dr. Atkinson was professor of materia medica at the University of Maryland for fourteen years. He was graduated from this university in 1865.

---

The use of an "invalid table," the shelf of which projects over the patient's body, will be found a great convenience during operations as a receptacle for instruments in immediate use. It saves time and temper, and avoids accumulation of instruments on the patient's body.—*American Journal of Surgery.*

---

The threading of catgut or kangaroo tendon through a needle-eye not very roomy may be made easy by cutting the suture end obliquely and flattening it between the handles of the scissors. Silk must not be cut obliquely, however, for this makes it apt to unravel while it is being threaded.—*American Journal of Surgery.*

---

That a bone appears normal by fluroscopic examination does not gainsay the presence of a fracture. A fracture of the radius, for example, may occur without displacement of the fragments. An x-ray plate will demonstrate the line of fracture, when the fluroscope fails to.—*American Journal of Surgery.*

---

When performing an office operation, too great care cannot be taken to sufficiently roll back or remove such articles o fclothing as might become soiled. The patient may not say much if he is obliged to draw up a garment wet with blood—but he'll probably think a few things.—*American Journal of Surgery.*

---

When removing a lipoma or other growth from the inner surface of the thigh, a little care should be exercised in order to avoid cutting the long saphenous vein. Ligature of that vessel (especially in ambulant and in non-aseptic cases) may be followed by a distressing phlebitis.—*American Journal of Surgery.*

---

Severe localized pain after traumatism, especially in children, may be due to subperiosteal fracture, *e. g.*, near the head of the humerus or the femur. Extreme localized tenderness is the chief sign; abnormal mobility and deformity are absent, and crepitus may not be elicited.—*American Journal of Surgery.*

---

An American physician who formerly practiced in El Paso, Texas, was recently convicted in Mexico, in conjunction with two other men, of having insured the lives of two men and murdering them to collect the insurance money. All of them have been sentenced to be shot, and the sentence has been confirmed by the supreme court.

---

Before performing curettage always make a final bimanual examination of the uterus in narcosis. The finding may determine some other form of treatment. Again, after curettage, before allowing the patient to get out of bed, carefully examine the pelvis for signs of a possible exudate.—*American Journal of Surgery.*

---

The admixture of adrenalin to cocain solution counteracts much of the depressant effect of the anesthetic and enhances the local vasoconstriction. When the mixture is used on the surface of a mucous membrane, however, as in excising an ulcer in the mouth, one must be prepared for a marked reactionary bleeding.—*American Journal of Surgery.*

---

A Mercier catheter is the first kind that ought to be employed in attempting to overcome retention caused by an enlarged prostate. Often it will have to be resorted to in the end; and, therefore, it will save much unsuccessful manipulation to use it at once. Occasionally, a metal catheter will pass when even a Mercier fails.—*American Journal of Surgery.*

---

Dr. Grant J. Ross, city physician of Sioux City, has given orders to stop any rummage sales projected by church societies and charitable institutions of the city, unless the second-hand clothing contributed for such sales first be disinfected. Dr. Ross says that several cases of contagious disease in Sioux City have been traced directly to goods purchased in rummage sales.

---

The proposal has been made in Ohio to organize a special section of the State Association for the Study of Pathology. One of the objects to be accomplished will be the establishing of a uniform technique in carrying out tests and other pathological work so that statistics gathered from different sections of the State may form a reliable basis for deductions.

---

Repeated attacks of "indigestion," not obviously due to some other condition, should awaken the suspicion of gall-stones. Most of the patients operated upon for cholelithiasis give a history of having been treated for a long time for "dyspepsia," and in many of these cases the correct diagnosis might earlier have been established.—*American Journal of Surgery.*

---

Dr. W. P. Spratling announces a prize of $500 offered by the Association for the Study of Epilepsy for the best essay on the eitlogy of epilepsy. The money is provided by persons interested in the work of the association. All essays submitted must be in English, must not contain more than 15,000 words, and must be sent to Dr. W. P. Spratling, Sonyea, N. Y., by September 1st, 1907.

---

With his left hand torn to pieces and his forearm crushed and firmly held between the rolls of a "fodder shredder," Dr. Charles McCullough, a well known young farmer and physician, who lives in Buckingham county, forty miles from Lynchburg, Va., cut his arm off below the elbow with his pocket knife. After freeing himself he directed the farm hands in taking up the several arteries, thus saving his life.

---

Permanent contracture of the muscles, notably of the flexor group in the forearm, may develop within a very short time after the application of a splint that exercises undue compression. It is a wise rule to inspect all fracture dressings within twenty-four hours; and when this is not expedient special care should be exercised, when applying the dressing, to avoid compression—*American Journal of Surgery.*

---

A bright and altogether satisfactory light for throat examinations can be had cheaply by covering a 16-candle-power Edison electric bulb with a smooth layer of plaster of Paris, about three-eighths of an inch thick, leaving on one side an aperture the size of a silver half-dollar, or larger. The white inner surface of the plaster brilliantly reflects the light. The other surface may be painted black for appearance's sake.—*American Journal of Surgery.*

---

If a patient gives a history of "sprained wrist" that has remained feeble and painful in spite of appropriate treatment for sufficient time, and if the wrist presents thickening and tenderness at its radial aspect, a diagnosis of fracture of the scaphoid should be entertained. Colles' fracture must be excluded, by the relation of the two styloid processes and the location of the deformity. Fractures of the radius and scaphoid may, however, coexist.—*American Journal of Surgery.*

---

The University of Georgia inaugurated a department of forestry Novemer 27th, with the following program:

Introductory Address . . . . . . . . . . . . . . . . Chancellor David C. Barrow

"The Progress of Forestry in the United States" . . . . . . . .

Mr. Alfred Gaskill,

Assistant Forest Inspector, U. S. Forest Service.

"Forestal Education" . . . . . . . . . . . . . . . . . . Professor Alfred Akerman

---

Everything is to be gained and nothing to be lost by having patients remove enough of their clothing, to allow of a completely satisfactory examination in all cases. Instances can be called to mind by any physician of erroneous judgments arrived at before exposure of other parts of the body showed conditions altering one's opinion. Especially is it important to compare the corresponding members of the body on the sound and the affected side, in all doubtful cases.—*American Journal of Surgery.*

At its recent meeting in Baltimore, the Southern Surgical and Gynecological Association selected New Orleans as the next place of meeting.

The following officers were elected:

President—Dr. Howard A. Kelly, of Baltimore.

Vice-Presidents—Dr. Rufus Fort, of Nashville, Tenn., and Dr. Hubert R. Royster, of Raleigh, N. C.

Secretary—Dr. William D. Haggard, of Nashville, Tennessee.

Treasurer—Dr. Charles M. Roser, of Dallas, Texas.

---

The Kings County Hospital Alumni Association has placed a bronze tablet 4x3 feet in the institution at Flatbush to the memory of the late Dr. Walter Reed, a former interne of the hospital. The tablet bears this inscription: "Erected by the Association of Ex-Internes of the Kings County Hospital to the memory of Walter Reed, M. D., interne in this hospital, 1871, Major and Surgeon, U. S. A., chairman United States Yellow Fever Commission, 1900-1901. He robbed the pestilence of its terrors and caused the cities of the Southland to sit in peace within their gates."

---

Occasionally, contractures of the fingers following the treatment of a cellulitis of the hand and forearm may be due, not to the cellulitis itself nor to the incisions made to relieve it, but to fibrosis and shortening of the flexors in the forearm, the result of too tight bandaging or strapping. Such a condition—Volkmann's ischemic muscle contracture—must, therefore, be distinguished from the stiff, flexed fingers produced by the cellulitis. Passive motions and massage are helpful in both conditions, but in the former bone shortening (radius and ulna) is necessary to accommodate the contractured muscles.—*American Journal of Surgery.*

---

A convenient way in which the anethesist may carry, all sterilized and ready for use, his hypodermatic solutions, is the following: Shallow, wide-mouthed, half-ounce bottles are sterilized, labeled and filled. Over the mouth of each bottle is then stretched, and hermetically fastened, a cover o fsterilized rubber (dam). Before the narcosis is begun the naesthetist disinfects his syringe and sets these bottles in a dish of sublimate solution. This sterilize the surface of the rubber. When a solution is wanted the needle of the hypodermatic syringe is simply thrust through the rubber and as much as is needed is drawn into the barrel. The puncture hole closes without leaage. The covers of the bottles need to be changed only occasionally.—*American Journal of Surgery.*

---

The annual meeting of the Mississippi Valley Medical Association was held at Hot Springs, Ark., November 6, 7 and 8, 1906. The following officers were elected:

President, H. Horace Grant, M. D., Louisville, Ky.
First Vice-President, G. A. Herbert, M. D., Hot Springs, Ark.
Second Vice-President, T. C. Witherspoon, M. D., St. Louis, Mo.
Secretary, Henry Enos Tuley, M. D., re-elected, Louisville, Ky.
Treasurer, S. C. Stanton, M. D., re-elected, Chicago, Ill.

Columbus, O., was selected as the next place of meeting, during October, 1907.

It was voted at this meeting to offer a prize of $100 to members of the Association for the best essay recording some original research work in the Mississippi Valley. A committee of three was appointed who will formulate rules of the contest, which will be published later.

---

"At the regular meeting of the Second Section of the American Urological Association, held in New York on Wednesday, October 24th, 1906. . . . . . .

"The President, Winfield Ayres, M. D., officially announced the death of the Vice-President of the Second Section, William K. Otis, and called for a report by the Committee appointed for the purpose, to present a memorial on the Association's bereavement. In presenting the report, a member of the Committee said:

"The ties of life-long intimacy which bound most of us to Dr. Otis, make his death a subject of grief to each individual. The usual set form of preamble and resolutions, therefore, were deemed inadequate by your Committee to express our sorrow. 'Billy's' demise is, to the older members of the Association as if a much loved brother had gone from us. Your Committee begs to submit:

William Kelly Otis' earthly career ended on Sept. 22nd, 1906.

To the members of the American Urological Association, his death is a threefold blow.

Most if us knew him intimately from his childhood; by his decease we lose a consistent friend, a charming companion, a most estimable colleague.

To the Science of Urology his death means an irreparable loss. Cut off in the midst of his career, his inventiev genius is stopped; the new and useful instruments he was continually devising must now be perfected by other hands. The advances in our work, he can no longer aid in developing.

The American Urological Association loses one of its founders, one of its most active coadjutors, one of its truest adherents.

Our Association shares with the family of William K. Otis, with the Profession at large, and with that world in which true manhood is understood and appreciated, that deep grief which the death of so noble a character inspires.

RAMON GUITERAS,
A. ERNEST GALLANT,
FERD. C. VALENTINE
Committee.

